# Membrane-permeabilized sonodynamic therapy enhances drug delivery into macrophages

**DOI:** 10.1371/journal.pone.0217511

**Published:** 2019-06-10

**Authors:** Zhengyu Cao, Tianyi Zhang, Xin Sun, Mingyu Liu, Zhaoqian Shen, Bicheng Li, Xuezhu Zhao, Hong Jin, Zhiguo Zhang, Ye Tian

**Affiliations:** 1 Department of Cardiology, the First Affiliated Hospital, Cardiovascular Institute, Harbin Medical University, Harbin, China; 2 Karolinska Institute, Department of Medicine, Stockholm, Sweden; 3 Laboratory of Photo- and Sono-theranostic Technologies and Condensed Matter Science and Technology Institute, Harbin Institute of Technology, Harbin, China; 4 Department of Pathophysiology and Key Laboratory of Cardiovascular Pathophysiology, Harbin Medical University, Key Laboratory of Cardiovascular Medicine Research (Harbin Medical University), Ministry of Education, Harbin, China; 5 Heilongjiang Academy of Medical Sciences, Harbin, China; Massachusetts General Hospital, UNITED STATES

## Abstract

Macrophages play a pivotal role in the formation and development of atherosclerosis as a predominant inflammatory cell type present within atherosclerotic plaque. Promoting anti-atherosclerotic drug delivery into macrophages may provide a therapeutic potential on atherosclerotic plaque. In this study, we investigated whether membrane-permeabilized sonodynamic therapy (MP-SDT) enhances drug delivery into THP-1 macrophages. Images of confocal microscopy confirmed that the optimal plasma distribution of the sonosensitizer protoporphyrin IX (PpIX) was at 1 hour incubation. The non-lethal parameter of MP-SDT was determined by cell viability as measured by a CCK-8 assay. Bright field microscopy demonstrated plasma membrane deformation in response to MP-SDT. Using SYTOX Green, a model drug for cellular uptake, we found that MP-SDT significantly induced membrane permeabilization dependent on ultrasound intensity and exposure time. Using Fluo-3 AM, intracellular calcium elevation during MP-SDT was confirmed as a result of membrane permeabilization. Membrane perforation of MP-SDT-treated cells was observed by scanning electron microscopy and transmission electron microscopy. Moreover, MP-SDT-induced membrane permeabilization and perforation were remarkably prevented by scavenging reactive oxygen species (ROS) during MP-SDT. Furthermore, we assessed the therapeutic effect of MP-SDT in combination with anti-atherosclerotic drug atorvastatin. Our results showed that MP-SDT increased the therapeutic effect of atorvastatin on lipid-laden THP-1-derived foam cells, including decreasing lipid droplets, increasing the cholesterol efflux and the expression of PPARγ and ABCG1. In conclusion, MP-SDT might become a promising approach to facilitating the delivery of anti-atherosclerotic drugs into macrophages via membrane permeabilization.

## Introduction

Atherosclerosis is a major contributor to the global burden of cardiovascular events, such as myocardial infarction and stroke [1]. Macrophages, a predominant inflammatory cell type present within atherosclerotic plaque, are critical to the formation and development of atherosclerosis and the progression and rupture of plaque [2]. Anti-atherosclerotic drugs that exert a lipid-efflux effect on macrophages resident in atherosclerotic plaque, like statins, can inhibit atherosclerotic plaque progression [3]. Enhancement of anti-atherosclerotic drug delivery into intraplaque macrophages will greatly promote therapeutic efficiency [4].

Increasing membrane permeability induces the passive diffusion of drugs into the cells, representing a promising strategy to facilitate drug delivery into target cells [5]. The pulsed electromagnetic field is known to induce the electroporation on plasma membrane allowing drugs to enter the viable cells [6]. Ultrasound in combination with microbubbles (USMB) increases cellular permeability in which ultrasound activates microbubbles to oscillate nearby cells and creates sonoporation [7]. These methods have been applied in anti-cancer chemotherapy [8, 9]. However, cell targeting of these techniques is limited, so that they may not be very effective for delivering drug into intraplaque macrophages. The technique that increases membrane permeability of intraplaque macrophages has not been reported and is highly desirable.

Sonodynamic therapy (SDT) is a novel strategy to exert synergistic effects by combining a sonosensitizer and ultrasound to induce various biological effects [10]. SDT has several advantages including cell targeting, uniform bioeffect and deep penetration [11, 12]. Protoporphyrin IX (PpIX) as a classical sonosensitizer has been reported to bind to lipid bilayer membrane, and produce a large amount of intracellular reactive oxygen species (ROS) at the sites where the sonosensitizer is localized upon ultrasonic activation [13]. Our previous study demonstrated that SDT selectively induces apoptosis of intraplaque macrophages [14]. However, it has not been reported whether PpIX-mediated SDT can promote drug delivery into macrophages. Here, we hypothesized that activating membrane-located PpIX by ultrasound, the membrane-permeabilized sonodynamic therapy (MP-SDT), facilitates drug delivery into macrophages.

In this study, we determined the non-lethal parameters of MP-SDT for macrophage viability and verified that MP-SDT promoted permeability of macrophages as measured by a model drug SYTOX Green and intracellular calcium probe Fluo-3 AM. We observed that MP-SDT induced membrane perforation and verified the perforation was dependent on ROS generation. Furthermore, we demonstrated that in combination of MP-SDT, the therapeutic effect atorvastatin, a well-known lipid-lowering drug, was significantly enhanced in THP-1-derived foam cells. Our work suggests that MP-SDT might become a potential technique to enhance delivery of anti-atherosclerotic drugs into macrophages.

## Materials and methods

### Chemicals

Phorbol-12-myristate-13-acetate (PMA), protoporphyrin IX (PpIX), PKH67 Green Fluorescent Cell Linker Kit, N-acetyl-L-cysteine (NAC), and verapamil were purchased from Sigma-Aldrich (St Louis, MO, USA). Fetal bovine serum (FBS) and RPMI-1640 were purchased from Hyclone Laboratories, Inc. (Logan, UT, USA). Fluo-3 AM, 2-7-dichlorofluorescin diacetate (DCFH-DA), and Hoechst 33342 were purchased from Beyotime Biotechnology (Beijing, China). 1,2-Bis (2-aminophenoxy) ethane-N,N,N′,N′-tetraacetic acid tetrakis (acetoxymethylester; BAPTA-AM) was purchased from Biolite Biotechnology (Tianjin, China). Ethylene glycol-bis(β-aminoethyl ether)-N,N,N',N'-tetraacetic acid (EGTA) was purchased from Leagene Biotechnology (Beijing, China). Oxidized-low-density lipoprotein (ox-LDL) was purchased from Yiyuan Biotechnologies (Guangzhou, China). RN1734 was purchased from Medchemexpress (Shanghai, China).

### Cell culture

Human THP-1 monocytes were obtained from American Type Culture Collection (Manassas, VA, USA). The cells were cultured in RPMI-1640 medium containing 10% fetal bovine serum at 37°C in a humidified atmosphere containing 5% CO_2_. For the THP-1 monocytes to differentiate into macrophages, the cells were seeded in 35-mm culture dishes or 96-well microplates at a density of 0.5 × 10^6^ cells/ml and treated with 100 ng/mL phorbol-12-myristate-13-acetate for 72 h. The THP-1 macrophages were subsequently transformed into foam cells by adding 50 μg/ml ox-LDL in serum-free RPMI 1640 medium for 18 h.

### Cell viability assay

The cell viability was assessed by the cell counting kit 8 (CCK-8) assay (Yeasen, Shanghai, China). Live cells were counted according to the optical density (OD) of each well which was quantified by an enzyme-linked immunosorbent assay microplate reader (Varian Australia Pty Ltd, Melbourne, VIC, Australia) at 450 nm. The OD of the results was indicated as the percentage of cell viability in the control group that was set as 100%.

### Plasma membrane localization of PpIX

THP-1-derived macrophages were incubated with serum-free RPMI 1640 medium containing 2 μM PpIX for 0.5, 1 and 4 h, followed by adding 1 μM PKH67 and 1 μM Hoechst 33342 for 5 min. After washing twice with phosphate-buffered saline (PBS), PpIX distribution was studied by a Zeiss LSM 700 confocal system (Zeiss, Germany).

### Membrane-permeabilized sonodynamic therapy

For the MP-SDT group, THP-1-derived macrophages or foam cells were incubated with serum-free RPMI 1640 medium containing 2 μM PpIX for 1 h. The control and ultrasound alone groups received an equivalent volume of medium without PpIX. After washing the residue of PpIX with PBS, the cells of the MP-SDT group were exposed to ultrasound wave at different intensities and sonication time. For the ultrasound group, the cells received ultrasound alone at the same settings. The ultrasound system employed was provided by the Condensed Matter Science and Technology Institute of the Harbin Institute of Technology (Harbin, China). As previously described [15], the parameters of homemade ultrasonic transducer were: diameter, 3.5 cm; resonance frequency, 1.0 MHz; duty factor, 10%; repetition frequency, 100 Hz. Acoustic intensities in the range of 0.5 W/cm^2^ (I_SATA_, spatial average time average intensity) were used depending on the apparatus. During the sonication procedure, the temperature of the solution inside the Petri dishes did not rise more than 0.1°C, as measured with a thermometer.

### Real-time detection for fluorescence signal

After being loaded with or without PpIX, cells were washed with PBS. For intracellular calcium and ROS detections, cells were loaded with 5 μM Fluo-3 AM for 30 min and 10 μM DCFH-DA for 30 min, respectively. For membrane permanent assessment, cells were incubated with 2 μM SYTOX Green. During ultrasound sonication, fluorescent signals were recorded by a fluorospectrophotometer at 3-second intervals over 4 minutes. Bright field images and fluorescent images were recorded at 1-minute intervals during ultrasound sonication. Cell size and fluorescent intensity were measured with Image-Pro Plus 6.0. To study extracellular calcium influx, cells were pre-incubated with 10 μM BAPTA-AM or 2mM EGTA. To study the role of L-type voltage-dependent calcium channel (L-VDCC) and transient receptor potential vanilloid 4 (TRPV4) in the intracellular calcium elevation, cells were pre-incubated with 10 μM verapamil and 10 μM RN1734 for 2 h before MP-SDT was administrated, respectively. To study the role of ROS in permeabilization, cells were pre-incubated with 5 mM NAC for 1 h before MP-SDT.

### Flow cytometric assay

Cells were washed with PBS and incubated with SYTOX Green. After different treatment (MP-SDT, ultrasound alone, PpIX alone and non-treatment), cells were immediately collected and resuspended in PBS, and analyzed by flow cytometry with BD FACSDiva Software v7.0 (Becton- Dickinson, USA).

### Electron microscopic assessment

Cells were processed for electron microscopy as previously described [16]. Scanning electron microscopy (SEM) specimens were imaged with a S-3400N SEM (HITACHI, Tokyo, Japan). Transmission electron microscopy (TEM) specimens were imaged with a JEM-1220 TEM (JEOL Ltd, Tokyo, Japan). Pores on cell membrane were calculated and measured with Image-Pro Plus 6.0 for each image.

### Oil red O staining

After transformed to foam cells, the macrophages were incubated with or without RPMI 1640 medium containing 10 μM atorvastatin. Then, the cells were treated with or without MP-SDT. After 24 h, the cells were fixed with 4% paraformaldehyde, followed by dehydration with 60% isopropanol for 2 min. Next, the neutral lipids were stained with filtered 0.3% oil red O (Jiancheng Bioengineering Institute, Nanjing, China) solution for 10 min at room temperature. Subsequently, hematoxylin was used to counterstain the cell nuclei for 1 min after rinsing with ddH_2_O. The oil red O stained lipid droplets were observed with light microscopy.

### Cholesterol efflux assay

The capacity of macrophage cholesterol efflux was measured using Cholesterol Efflux Fluorometric Assay Kit (BioVision, Inc., USA) by following the manufacturer’s instructions. After treatment, foam cells in 96-well plate (black plate) were incubated with fluorescence-labeled cholesterol for 16 h at 37 °C. Then, cells were treated with cholesterol acceptors high-density lipoprotein (50 μg/well). Six hours later. After 6 h treatment, the supernatant and cell monolayer solubilized with cell lysis buffer were transferred to a 96-well plate (white plate) and measured at 482/515 nm. The cholesterol efflux is calculated as follows:

Cholesterolefflux%=Fluorescenceintensityofthemedia/(Fluorescenceintensityofthecelllysate+media)×100.

### Western blot analysis

After respective treatments as indicated in individual experiments, the cells were lysed in RIPA buffer containing protease and phosphatase inhibitors on ice. After quantification and denaturation, equal amounts of protein samples were electrophoresed in SDS-polyacrylamide gel and transferred onto PVDF membranes (Millipore, Schwalbach, Germany), followed by blocking for 2 h at room temperature with 5% dried skimmed milk in Tris-buffered saline with 0.05% Tween 20. The membranes were probed with specific primary antibodies for β-actin (ABclonal Technology, Wuhan, China), ATP binding cassette subfamily G member 1 (ABCG1), ATP binding cassette subfamily A member 1 (ABCA1) and peroxisome proliferator-activated receptor γ (PPARγ) (Abcam, Cambridge, UK) at 4 °C overnight with slight agitation and then incubated with Horseradish peroxidase (HRP)-conjugated secondary antibodies for 2 h at room temperature. The membranes were visualized by chemiluminescence method using chemiluminescence detection kit (Haigene, China) on a Bio-Rad ChemiDocTM MP Imaging System (California, USA).

### Statistical analysis

All of the experiments described were performed independently at least three times. Data are presented as the mean ± standard deviation (SD). Data among the treatment groups were assessed with one-way or two-way ANOVA analysis performed in GraphPad Prism (La Jolla, CA, USA). Statistical significance is defined as p-value < 0.05.

## Results

### Parameters of MP-SDT

**Fig 1:**
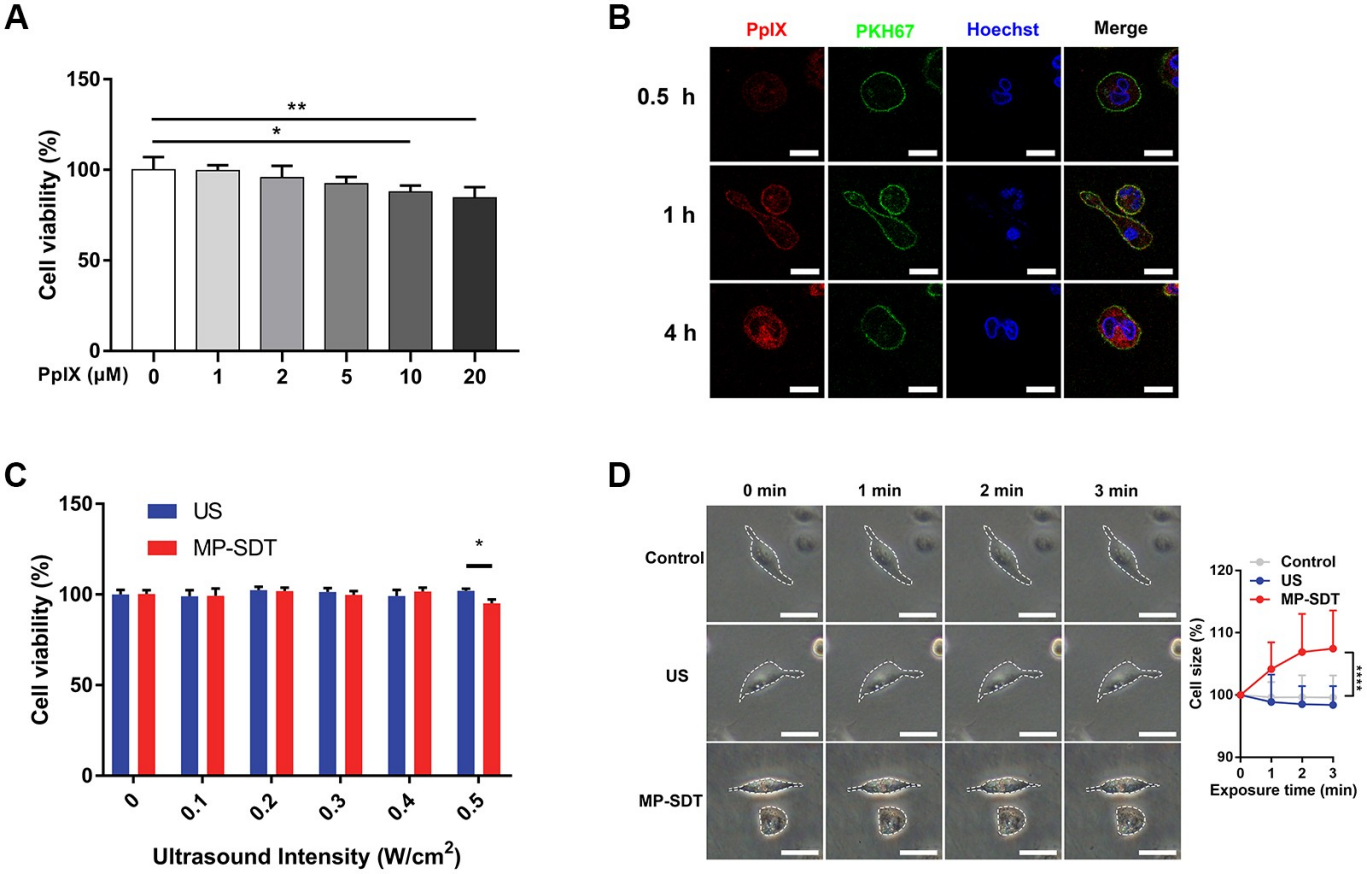
Parameters of MP-SDT. (A) Cell viability of THP-1 macrophages after incubation with different concentrations of PpIX. (B) Representative confocal fluorescent images of PpIX distribution at different time points. Scale bar = 20 μm. (C) Cell viability after MP-SDT at different ultrasonic intensity. (D) Representative images of cell morphology change and relative cell size change analyzed with phase-contrast microscopy. Dashed line indicates cell profile. Scale bar = 50 μm. US: ultrasound. Data are shown as mean ± SD. *p<0.05, **p<0.01, ****p<0.0001.

To ensure that the application of exogenous PpIX alone does not affect the survival of THP-1 macrophages, different doses of PpIX incubation were examined. We determined that 2 μM PpIX did not significantly affect cell survival (Fig 1A). To determine the incubation time for the optimal distribution of PpIX on cell membrane, we used membrane probe PKH67 to study the co-localization of PpIX and plasma membrane for different PpIX incubation times. After 1 h incubation, the PpIX was well distributed on plasma membrane co-localized with PKH67 (Fig 1B). Next, we evaluated cell viability after MP-SDT with 1-hour incubation of 2 μM PpIX at different ultrasonic intensity. Ultrasonic intensity of 0.4 W/cm^2^ did not cause a significant decrease in cell survival (Fig 1C). In order to avoid cell death, we chose 0.4 W/cm^2^ in the following experiments. We then examined the change of cell morphology during sonication, and found that MP-SDT-induced cellular enlargement mainly occurred within the first 2 min (Fig 1D). Taken together, our results showed the parameters of non-lethal MP-SDT as 1-hour incubation of 2 μM PpIX and 0.4W/cm^2^ and 2 min of sonication.

### MP-SDT induces SYTOX Green uptake by macrophages in ultrasound-dependent manner

**Fig 2:**
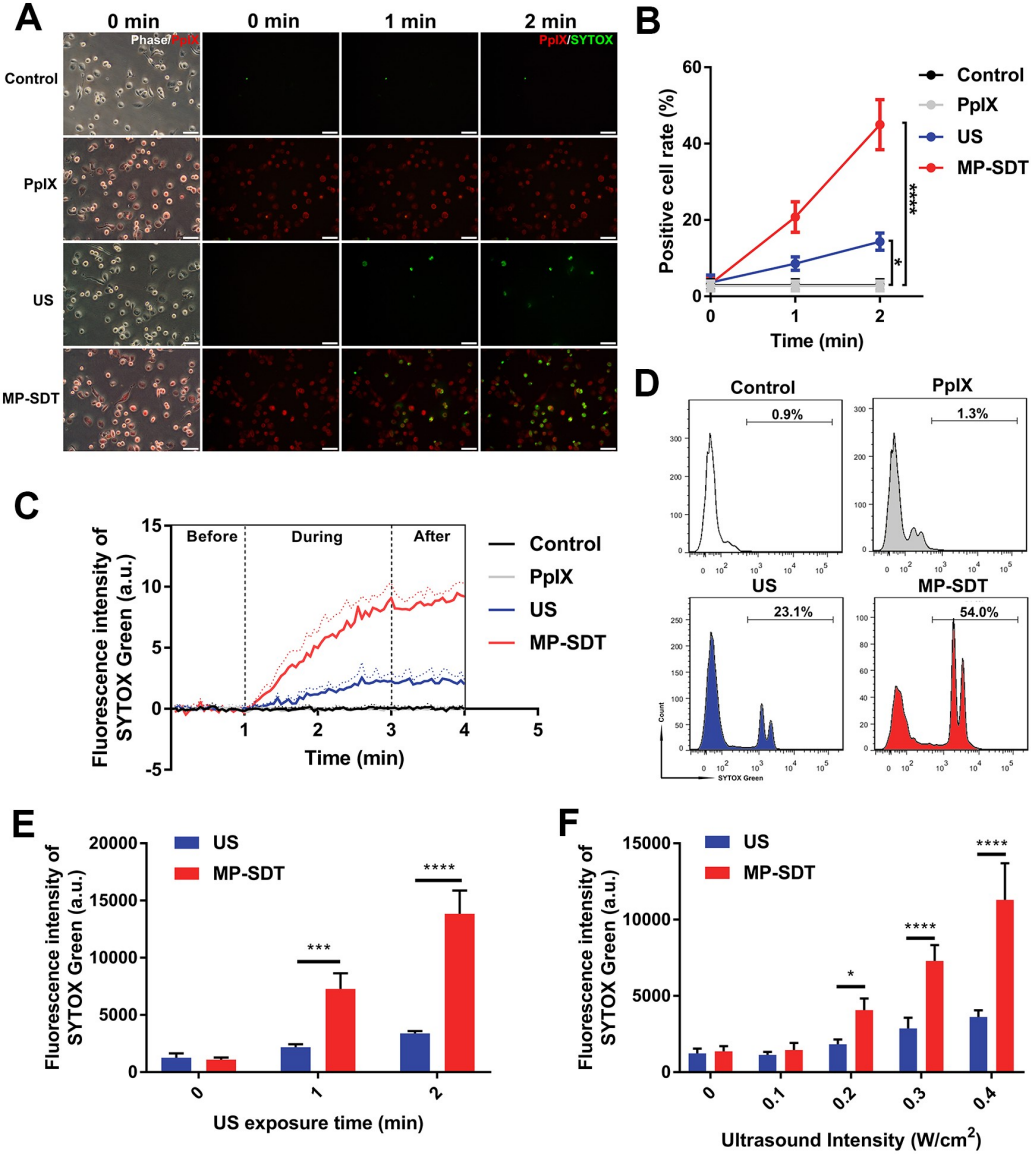
MP-SDT induces drug uptake by macrophages in ultrasound-dependent manner. (A) Representative images of SYTOX Green uptake of the control, PpIX, ultrasound and MP-SDT groups. Scale bar = 50 μm. (B) SYTOX Green positive cell ratio in different groups at indicated time point. (C) Real-time detection of SYTOX Green fluorescence by a fluorospectrophotometer. (D) SYTOX Green fluorescence measured by flow cytometry. (E and F) SYTOX Green fluorescence at different ultrasound intensity and exposure time. US: ultrasound. Data are shown as mean ± SD. *p<0.05, ***p<0.001, ****p<0.0001.

When cells are permeabilized, membrane impermeant compounds will transport into the cytoplasm [17]. Therefore, we estimated the permeabilization efficacy with impermeable model drug SYTOX Green which will exhibit a green fluorescent signal when entering and binding to nucleic acids [18]. Fluorescent microscopy showed that cells of the control group and the PpIX group cannot be stained with SYTOX Green with an intact membrane (Fig 2A). Upon exposed to MP-SDT, many cell membranes were permeabilized, leading to an increased number of green fluorescent cells. SYTOX Green positive rate of the MP-SDT group was significantly higher than that of the ultrasound group (p<0.0001, Fig 2B). Similarly, real-time detection of the fluorescence intensity of SYTOX Green showed a significant increase during MP-SDT compared with that in the ultrasound alone group (Fig 2C). The result of flow cytometry further confirmed that the SYTOX Green uptake was obviously increased by MP-SDT, whereas only slightly increased by ultrasound (Fig 2D). MP-SDT-induced drug uptake was dependent on ultrasound exposure time and intensity (Fig 2E and 2F).

### MP-SDT induces intracellular calcium elevation

**Fig 3:**
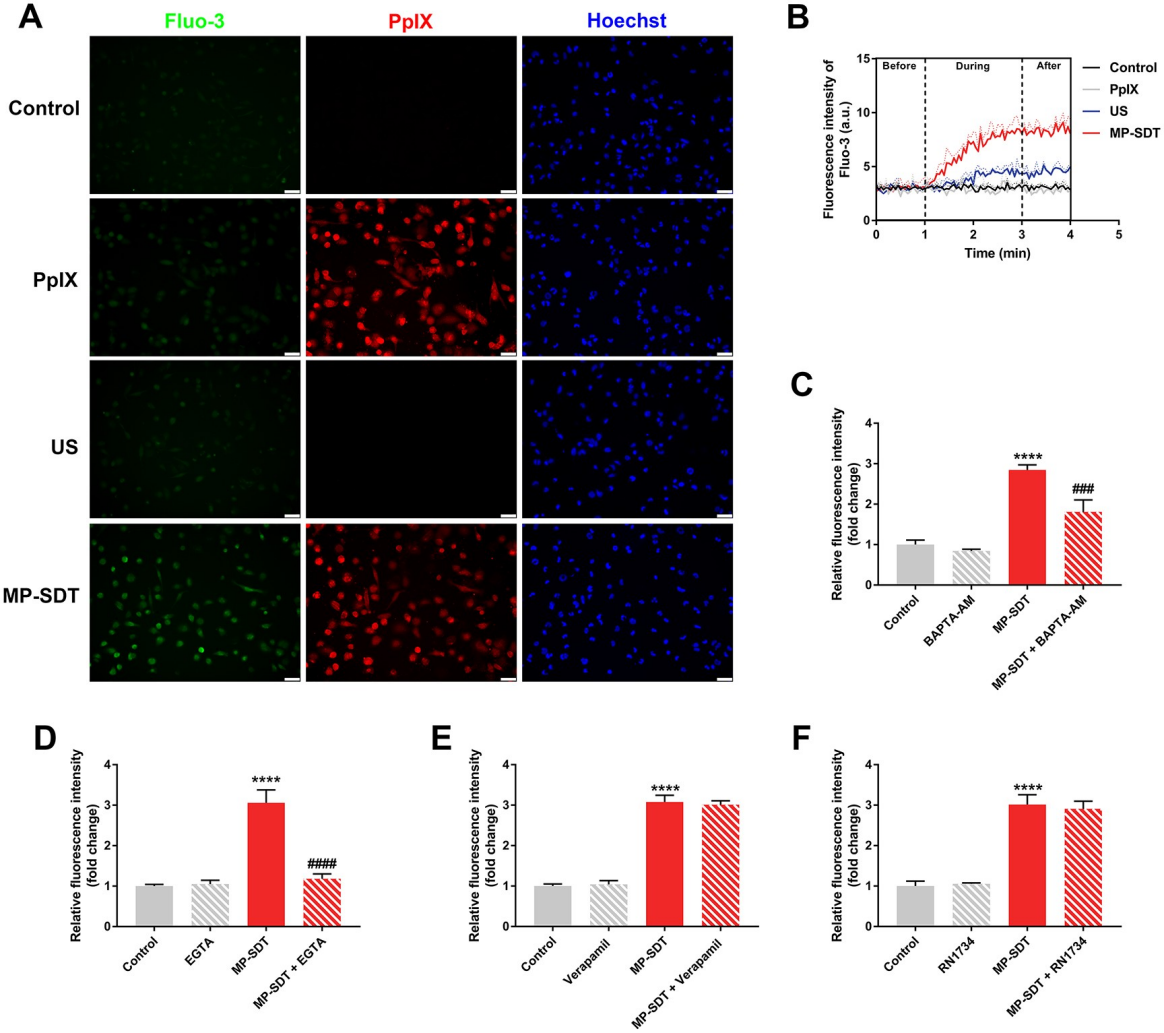
MP-SDT induces intracellular calcium elevation. (A) Representative fluorescence images of fluorescence of intracellular Fluo-3 in the control, PpIX, ultrasound and MP-SDT group. Scale bar = 50 μm. (B) Real-time detection of fluorescence intensity of intracellular Fluo-3 change in different groups. (C-F) The effect of BAPTA-AM (C), EGTA (D), verapamil (E) and RN1734 (F) on intracellular calcium elevation during MP-SDT. US: ultrasound. Data are shown as mean ± SD. ****p<0.0001 vs. control; ###p<0.001, ####p<0.0001 vs. SDT.

As an important signaling ion, calcium is controlled by plasma membrane in the cytosol at submicromolar (μM) concentration whereas the extracellular Ca^2+^ concentration is generally in a range of a few mM [19]. Therefore, we investigated whether extracellular calcium influx occurs during MP-SDT. The fluorescence intensity of Fluo-3, an intracellular calcium probe, obviously increased after MP-SDT compared with that of the control group and ultrasound group, assayed with fluorescence images and real-time detection (Fig 3A and 3B). The MP-SDT-induced calcium increase was significantly inhibited by intracellular calcium chelator BAPTA-AM and extracellular calcium chelator EGTA, respectively (Fig 3C and 3D). Furthermore, two classical plasma calcium channels, L-type voltage-dependent calcium channel (L-VDCC) and transient receptor potential vanilloid 4 (TRPV4), were studied in this process. Both inhibitors of them had no effect on increased calcium during SDT (Fig 3E and 3F). These results indicated that MP-SDT-induced extracellular calcium influx may not be mediated by a specific calcium channel.

### MP-SDT induces plasma membrane perforation

**Fig 4:**
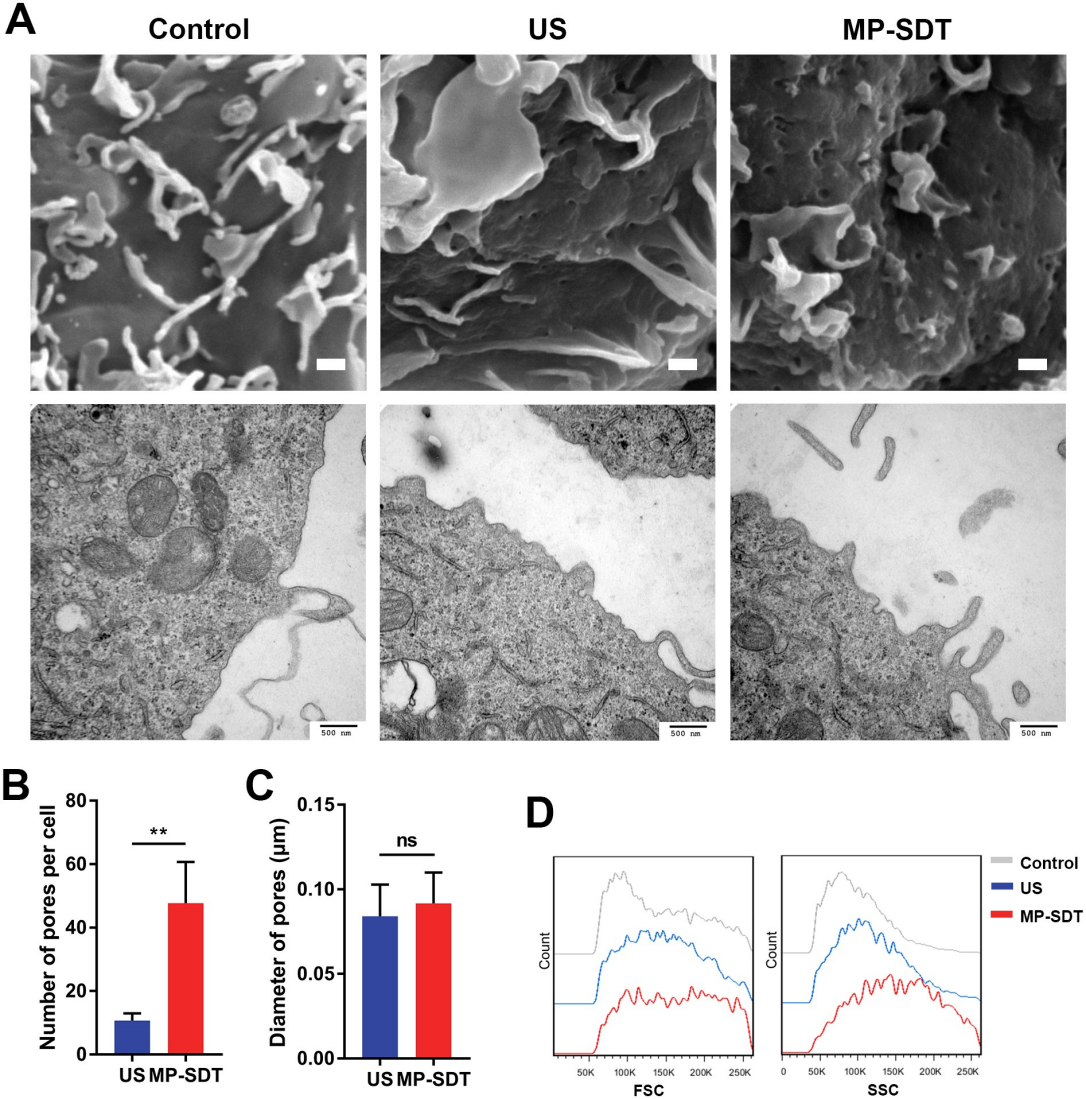
MP-SDT induces plasma membrane perforation. (A) Representative SEM and TEM images of the control, ultrasound and MP-SDT groups. Upper panel scale bar = 5 μm. Lower panel scale bar = 500 nm. (B) Quantitative assay of pore number per cell in the ultrasound and MP-SDT groups by SEM images. (C) Quantitative assay of pore diameters in the ultrasound and MP-SDT group by SEM images. (D) Representative histograms of the front scatter signal (FSC) and the side scatter signal (SSC) of macrophages in indicated groups were analyzed by flow cytometry. US: ultrasound. Data are shown as mean ± SD. ns: not significant, **p<0.01.

Next, we investigated the membrane morphology change after MP-SDT. Images of SEM and TEM showed pores on plasma membranes of the US and MP-SDT groups, whereas plasma membranes of the control group were even and intact. The images showed pores of the MP-SDT group were denser than those of the ultrasound group (Fig 4A). Quantification assay showed that there were more pores on plasma membranes of the MP-SDT group than on those of the ultrasound group (47.67 ± 13.05 vs. 10.67 ± 2.31, p<0.01, Fig 4B). The mean diameters of pores in the ultrasound group and the MP-SDT group were 84.05 ± 18.75 nm and 91.75 ± 18.19 nm, respectively, which were not significant different (Fig 4C). Moreover, the front and the side scatter signals detected by flow cytometer were increased in MP-SDT-treated macrophages (Fig 4D). These results further indicated irregularity of cellular membrane and cellular enlargement after MP-SDT treatment.

### MP-SDT induces intracellular ROS generation

**Fig 5:**
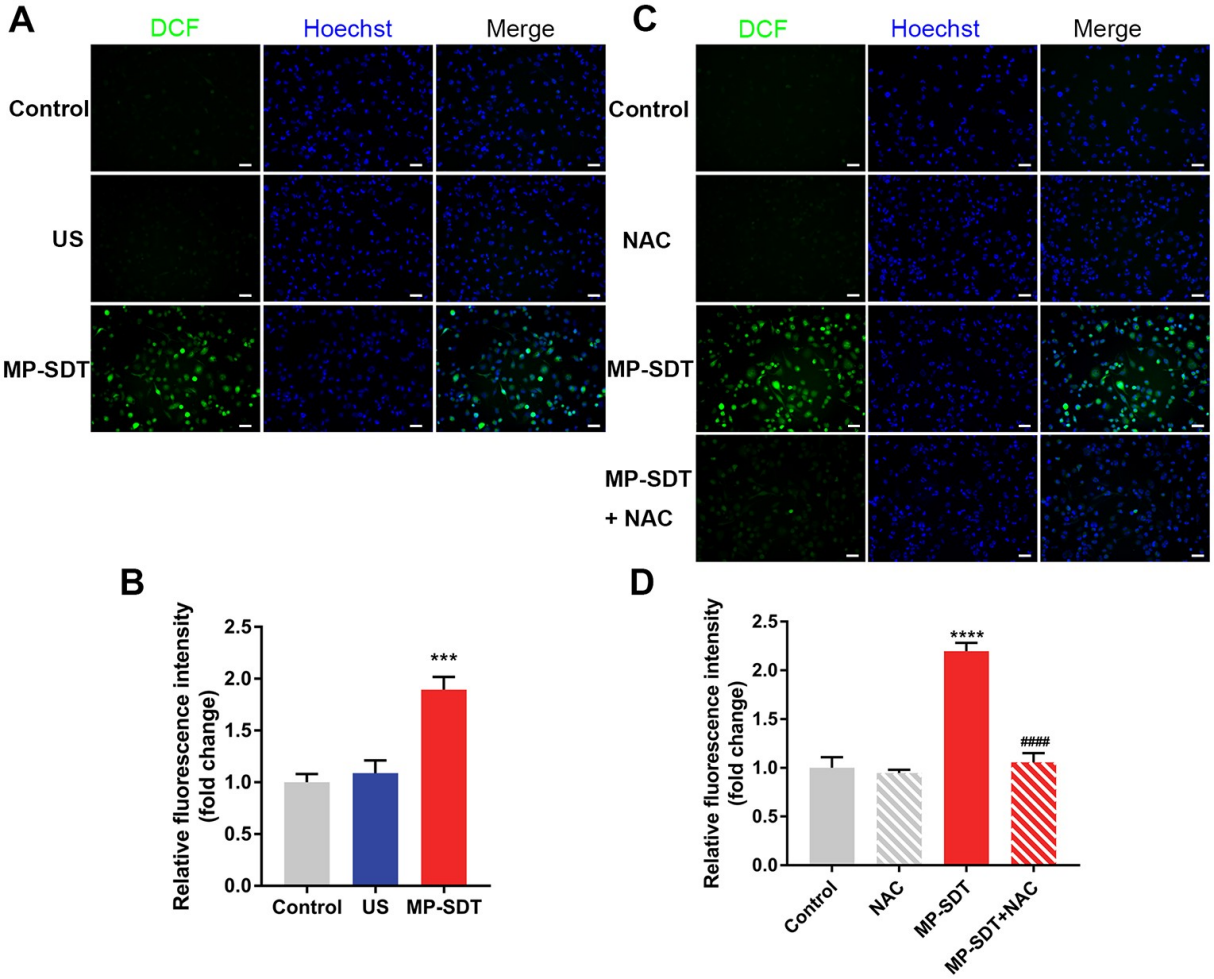
MP-SDT induces intracellular ROS generation. (A) Representative fluorescence images of fluorescence of DCF in the control, ultrasound and MP-SDT groups. Scale bar = 50 μm. (B) Relative fluorescence intensity of DCF of indicated groups. (C) Representative fluorescence images of untreated macrophages (control) and macrophages treat with NAC, MP-SDT, or MP-SDT + NAC. Scale bar = 50 μm. (D) Relative fluorescence intensity of DCF of indicated groups. US: ultrasound. Data are shown as mean ± SD. ***p<0.001, ****p<0.0001 vs. control, ####p<0.0001 vs. MP-SDT.

ROS generation is a critical factor in conventional SDT-induced cell apoptosis [20]. Therefore, we measured the intracellular ROS by DCFH-DA to explore whether ROS are generated in MP-SDT treatment. Fluorescent images of macrophages showed that the green fluorescence of DCF was slightly increased in the ultrasound group whereas it was obviously increased in the MP-SDT group (Fig 5A). The relative fluorescence intensity was significantly higher in MP-SDT-treated, than in ultrasound-treated cells (Fig 5B). Following pre-incubation with the ROS scavenger NAC, the fluorescence increase in the MP-SDT group was significantly prevented (Fig 5C and 5D).

### ROS scavenger inhibits MP-SDT-induced perforation and membrane permeabilization

**Fig 6:**
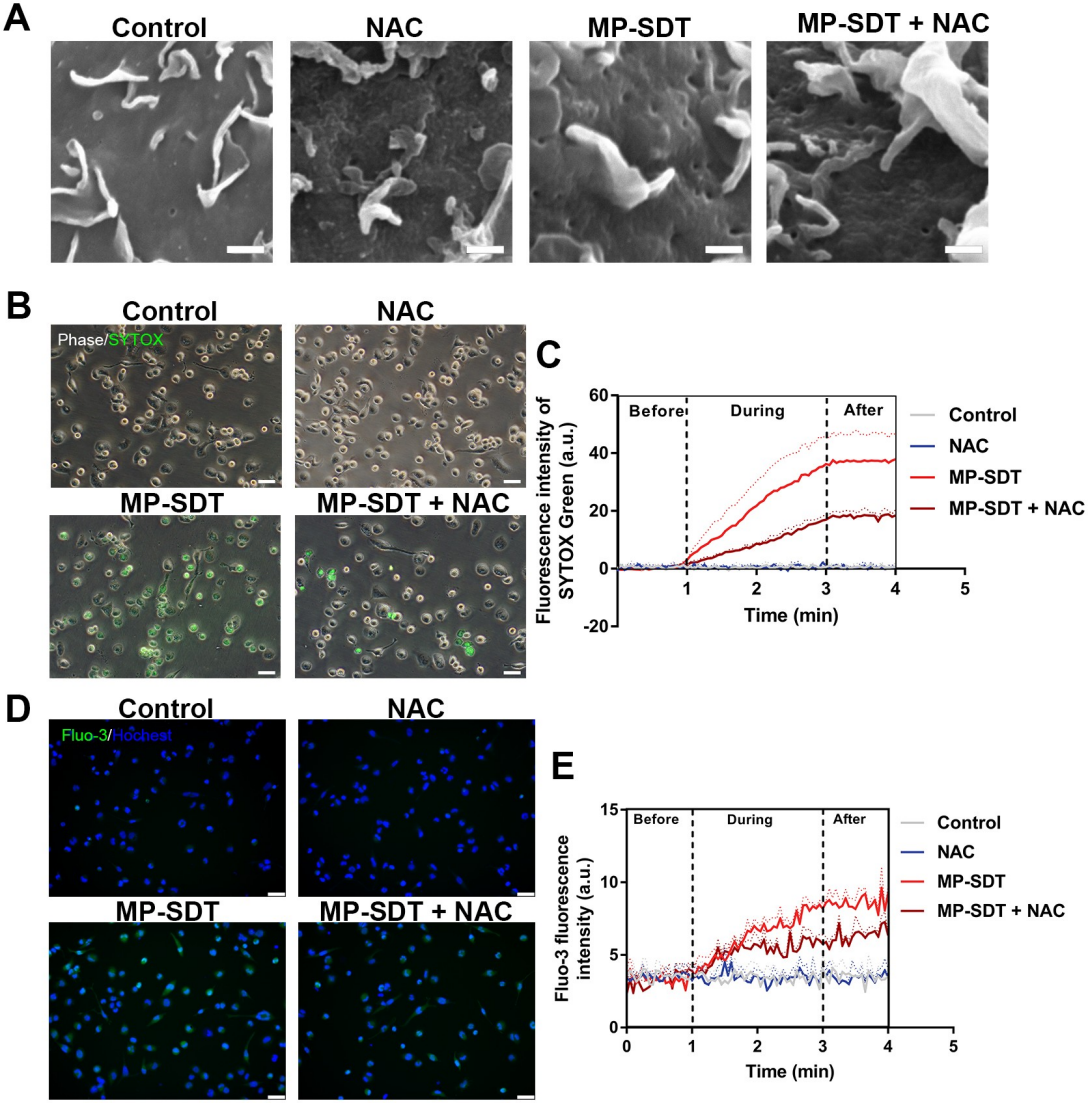
ROS scavenger inhibits MP-SDT-induced perforation and membrane permeabilization. (A) Representative SEM images of untreated macrophages (control) and macrophages treat with NAC, MP-SDT, or MP-SDT + NAC. Scale bar = 5 μm. (B) Representative fluorescence images of SYTOX Green uptake in indicated groups. Scale bar = 50 μm. (C) Real-time detection of fluorescence intensity of SYTOX Green uptake in different groups. (D) Representative fluorescence images of Fluo-3 in indicated groups. Scale bar = 50 μm. (E) Real-time detection of fluorescence intensity of Fluo-3 in different groups. Data are shown as mean ± SD.

To further explore the role of ROS in MP-SDT-induced membrane permeabilization, we observed cellular membrane by SEM and measured SYTOX Green uptake and intracellular calcium after pretreatment of ROS scavenger NAC. Images of SEM showed that MP-SDT-induced membrane perforation was obviously prevented by NAC (Fig 6A). In addition, SYTOX Green uptake of the MP-SDT group were significantly inhibited in the MP-SDT group pretreated with NAC compared to the MP-SDT group (Fig 6B and 6C). Similarly, intracellular calcium elevation was obviously suppressed in the MP-SDT + NAC group (Fig 6D and 6E).

### MP-SDT enhances the therapeutic effect of atorvastatin

**Fig 7:**
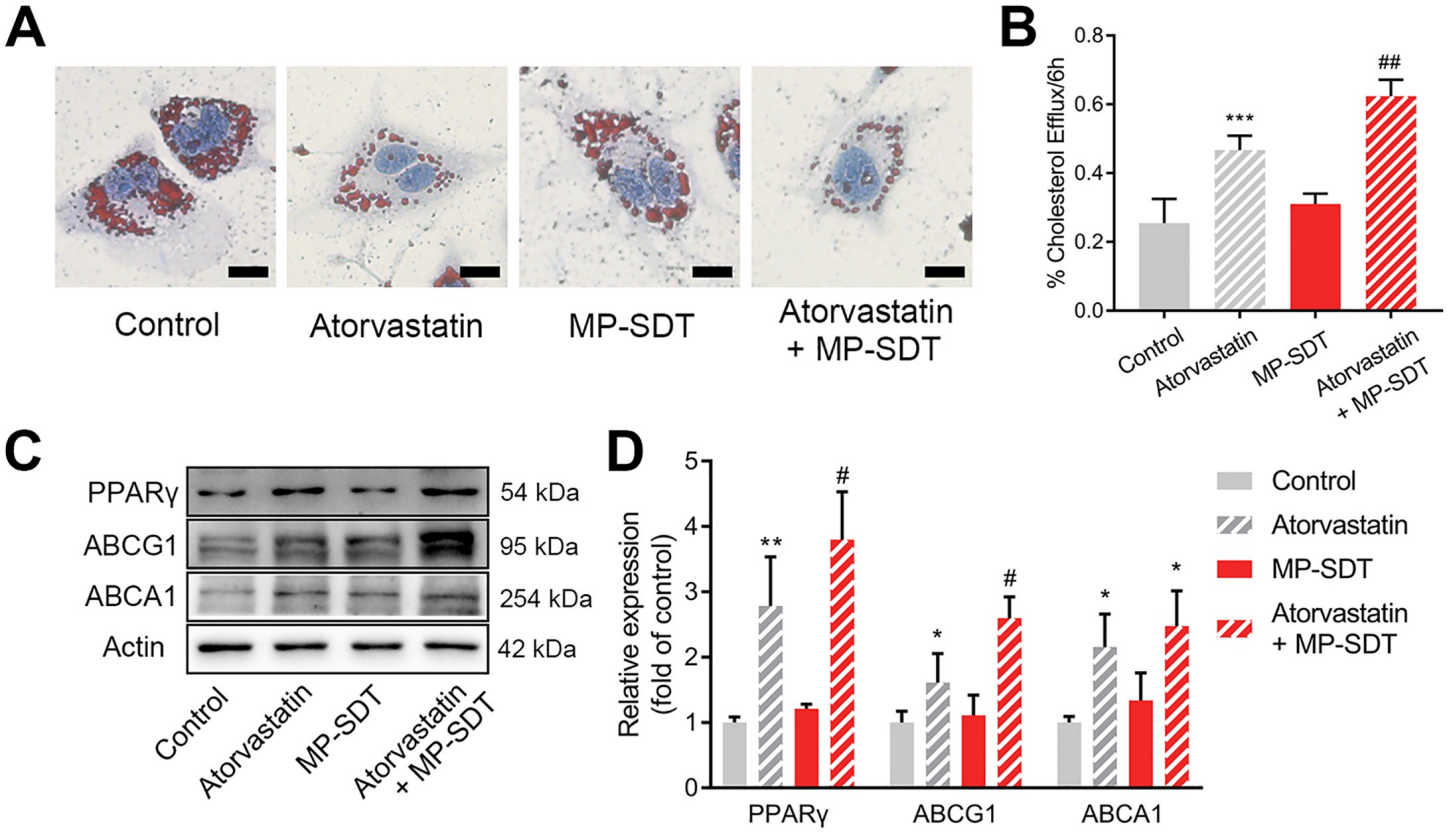
MP-SDT enhances the therapeutic effect of atorvastatin. (A) Representative images of THP-1-derived foam cells in control, atorvastatin, MP-SDT and atorvastatin combining MP-SDT group detected by Oil Red O staining. Scale bar = 20 μm (B) The percentage of cholesterol efflux in indicated groups. (C) Representative western blot of PPARγ, ABCG1 and ABCA1 expression in indicated groups. (D) Quantification of the fold change of indicated proteins. Data are shown as mean ± SD. *p<0.05, **p<0.01, ***p<0.001, ****p<0.0001 vs. control, #p<0.05, ##p<0.01 vs. atorvastatin.

Finally, we investigated whether MP-SDT can enhance the effect of atorvastatin in THP-1-derived foam cells. First, we confirmed the effect of permeabilization in MP-SDT-treated THP-1-derived foam cells (S1 Fig). Next, foam cells were randomly divided into four groups: control, atorvastatin alone, MP-SDT and atorvastatin + MP-SDT. Oil red O staining showed that intracellular lipid droplets were decreased in the atorvastatin group and atorvastatin + MP-SDT group (Fig 7A). Intracellular cholesterol efflux fluorometric assays verified that atorvastatin + MP-SDT significantly increased the percentage of cholesterol efflux compared with atorvastatin alone (Fig 7B). Cholesterol efflux related protein PPARγ, ABCG1 and ABCA1 were examined with the western blotting assay. Our results showed that the atorvastatin + MP-SDT group had a significant increase in the expressions of PPARγ and ABCG1 compared with those treated with atorvastatin alone (Fig 7C and 7D).

## Discussion

In this study, we investigated the effect of non-lethal MP-SDT combining the plasma membrane-located sonosensitizer PpIX and ultrasound at low intensity and short duration. We demonstrated that MP-SDT enhanced drug delivery into macrophages. We also found that MP-SDT-induced plasma membrane permeabilization is associated to ultrasound intensity and exposure time and is dependent on ROS production.

SDT, a novel noninvasive approach, involves a synergistic effect of sonosensitizer and ultrasound, leading to activation of cell autophagy, apoptosis or necrosis [21]. As a classical and extensively studied sonosensitizer, PpIX is initially localized in the plasma membrane and accumulated gradually into cytoplasm when the incubation time is prolonged [22, 23]. Previous studies have reported that conventional SDT induces cell apoptosis when sonosensitizer accumulates at mitochondria after a long incubation period [24]. In this study, at the time when PpIX was mainly distributed on plasma membrane, we found that ultrasound induces membrane deformation and permeabilization of macrophages. The intensity of MP-SDT (0.4 W/cm^2^ of ultrasonic intensity and 2 minutes of sonication time) was milder than that induced cell apoptosis [25, 26] and was non-lethal for macrophages (S2 Fig), since intracellular ROS production might be not enough to activate cell apoptosis. Therefore, our results indicate that MP-SDT-induced membrane permeabilization was not due to cell death and might be reversible [27].

Measuring intracellular uptake of the model drug SYTOX Green is an established method to evaluate membrane permeabilization [28]. Here, we found that MP-SDT caused about 50% macrophages permeabilized which was about 2-fold of that in the ultrasound group. In addition, MP-SDT-induced membrane permeabilization was dependent on ultrasound intensity and exposure time. Studies have reported that transient pores that created on the plasma membrane can increase cell permeability [29] However, whether the pores are created upon MP-SDT treatment has not been reported up to date. Here, we observed the membrane perforation in macrophages treated with MP-SDT. We believe that MP-SDT creates the pores on the membranes of macrophages, and thereby enhances the penetration of external substances into the cells [12]. The pores of each cell in the MP-SDT group were about 4.7 folds more than those in the ultrasound group, whereas the mean diameters were not significantly different. USMB induces a 3-fold increase of the mean number of pores per cell compared with ultrasound alone, and the mean diameter is ranging from several tens to a few hundred nanometers [30, 31]. Thus, MP-SDT may be an efficient technique that creates uniform pores on plasma membrane.

Plasma membrane permeabilization can induce influx of extracellular calcium into the cytoplasm because of the large concentration gradient across the cell membrane [32]. Our results showed that intracellular calcium of MP-SDT group significantly increased compared with that in the ultrasound alone group and the control group. Moreover, the calcium influx was neither through L-VDCC nor TRPV4 which is a mechanosensitive channel. We assumed that MP-SDT-induced calcium influx might be mainly through non-selective pores rather than ion channels. In fact, there are many other types of mechanosensitive ion channels in the plasma reported to be activated by ultrasound, such as the Piezo and K2P families, which needs further investigation to clarify [33].

ROS generation plays a pivotal role in SDT-induced cytoskeletal disruption and mitochondrial damage et al [25, 34]. When PpIX is activated by ultrasound, a large amount of ROS is produced for the cellular effects on the sites where PpIX is distributed [35]. In our study, intracellular ROS generation was also detected in macrophages after MP-SDT treatment. Previous study demonstrated that SDT-induced ROS oxidize membrane lipid and damage cellular membrane [36]. Likewise, ROS scavenger NAC remarkably suppressed the MP-SDT-induced permeabilization and perforation, suggesting that ROS generated on plasma membrane leads to nanoscale membrane disruption [12]. In addition, other factors such as mechanical stress might also be a potential mechanism of MP-SDT. Studies have reported that physicochemical properties of bilayer lipid membranes are altered after binding with PpIX [37]. The modified membranes become more vulnerable and hypersensitive to ultrasound [11], and more easily to form pores in the presence of an electrical field [38]. Therefore, the exact mechanisms of MP-SDT are complex and still need further investigation.

Vascular-resident macrophages take up oxidized lipids and become highly inflammatory, contributing to the progression and rupture of plaque [39]. Increasing their ability of lipid metabolism and cholesterol efflux can inhibit plaque progression and increase plaque stability [40]. Therefore, promoting cholesterol efflux targeting intraplaque macrophages is considered as a therapeutic strategy in the treatment of atherosclerosis [4]. Atorvastatin, one of 3-hydroxy-3-methylglutaryl coenzyme A (HMG-CoA) reductase inhibitors, is widely used to lower atherogenic lipoproteins by decreasing cholesterol biosynthesis in hepatocyte [41]. In addition, atorvastatin has a direct effect on macrophages [42] by promoting cholesterol efflux via activation of peroxisome proliferator-activated receptor γ (PPARγ), a key regulator of lipid metabolism, leading to the reduction of lipid burden and inhibition of plaque progression [43]. A previous study reported that facilitating the delivery of statins to intraplaque macrophages can decrease plaque area and inhibit plaque rupture [44]. Another study showed that the statin-loaded nanoparticle that allow for drug delivery to intraplaque macrophages suppresses plaque inflammation in apoE-KO mice [45]. In the present study, we demonstrated that MP-SDT promoted drug uptake in macrophage-derived foam cells and enhanced the effect of atorvastatin including increasing cholesterol efflux and the expression of PPARγ and ABCG1. This result suggests that MP-SDT might be able to increase the therapeutic efficacy of anti-atherosclerotic medications on atherosclerotic plaques by promoting drug uptake by intraplaque macrophages.

Ultrasound-targeted microbubble destruction (UTMD) has shown to be particularly promising for enhancing gene or drug delivery [46]. However, one critical issue is that expansions and collapses of microbubbles can induce endothelia erosion and exacerbate development of atherosclerotic plaque even leading to plaque rupture [47]. Our previous study showed that endothelial cells were intact on the plaque surface after SDT, since the sonosensitizer was only distributed in intraplauqe macrophages [48]. Additionally, limb perfusion and resistance index were similar before and after SDT indicating that SDT did not cause rupture of atherosclerotic plaques [49]. Therefore, MP-SDT might potentially be more appropriate for the treatment of atherosclerosis.

In summary, we have demonstrated that MP-SDT can facilitate drug delivery into THP-1 macrophages by permeabilization. MP-SDT might become a potential technique to increase efficacy of anti-atherosclerotic drugs via promoting drug delivery into intraplauqe macrophages.

## Supporting information

S1 Fig Effect of MP-SDT on THP-1-derived foam cell. (A) Representative images of PpIX in THP-1-derived foam cells. Scale bar = 50 μm. (B) Cell viability of THP-1-derived foam cells after non-treatment (control) and MP-SDT. (C) Relative fluorescence intensity of SYTOX Green in foam cells after non-treatment (control) and MP-SDT. Data are shown as mean ± SD. n.s, not significant, **p<0.01, vs. control.
(TIF)

S2 Fig Evaluation of cytotoxicity of MP-SDT. (A) Cell viability detected by Annexin V/PI flow cytometry analysis and quantitative assay. Annexin V-/PI- represents the viable cells. Annexin V+/PI- and Annexin V+/PI+ represent the apoptotic cells. (B) Representative western blots and relative quantitation of cleaved-caspase 9/pro-caspase 9 and cleaved-caspase 3/pro-caspase 3 after MP-SDT. ns: no significance.
(TIF)

S1 File Experimental data. (ZIP)
