# Supplementary figures and images for: Membrane-permeabilized sonodynamic therapy enhances drug delivery into macrophages
Source: PLoS One. 2019 Jun 10;14(6):e0217511. doi: 10.1371/journal.pone.0217511 (PMC6557485; doi:10.1371/journal.pone.0217511)

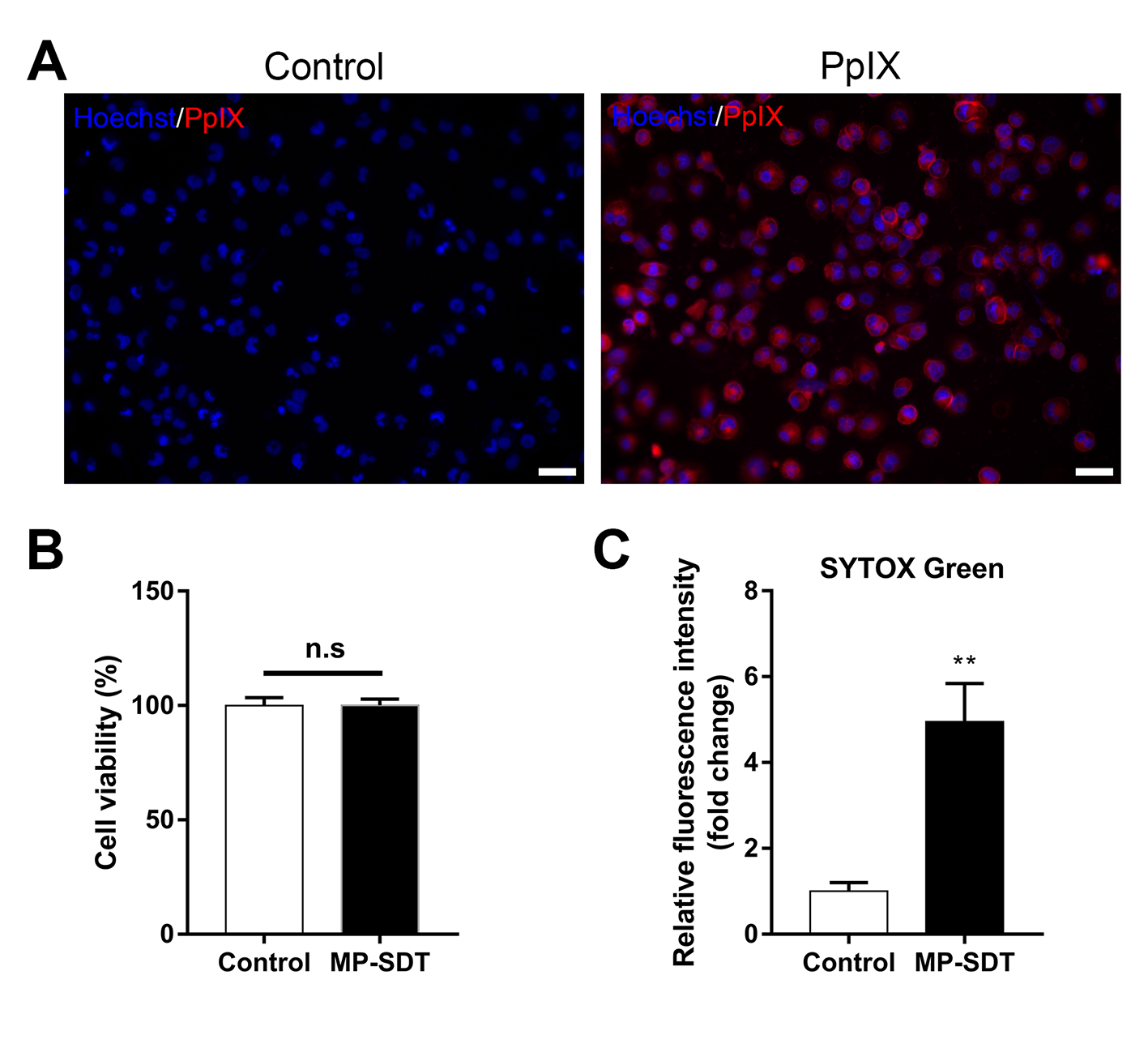

Supplement: S1 Fig — (A) Representative images of PpIX in THP-1-derived foam cells. Scale bar = 50 μm. (B) Cell viability of THP-1-derived foam cells after non-treatment (control) and MP-SDT. (C) Relative fluorescence intensity of SYTOX Green in foam cells after non-treatment (control) and MP-SDT. Data are shown as mean ± SD. n.s, not significant, **p<0.01, vs. control. (TIF) [file pone.0217511.s001.tif]

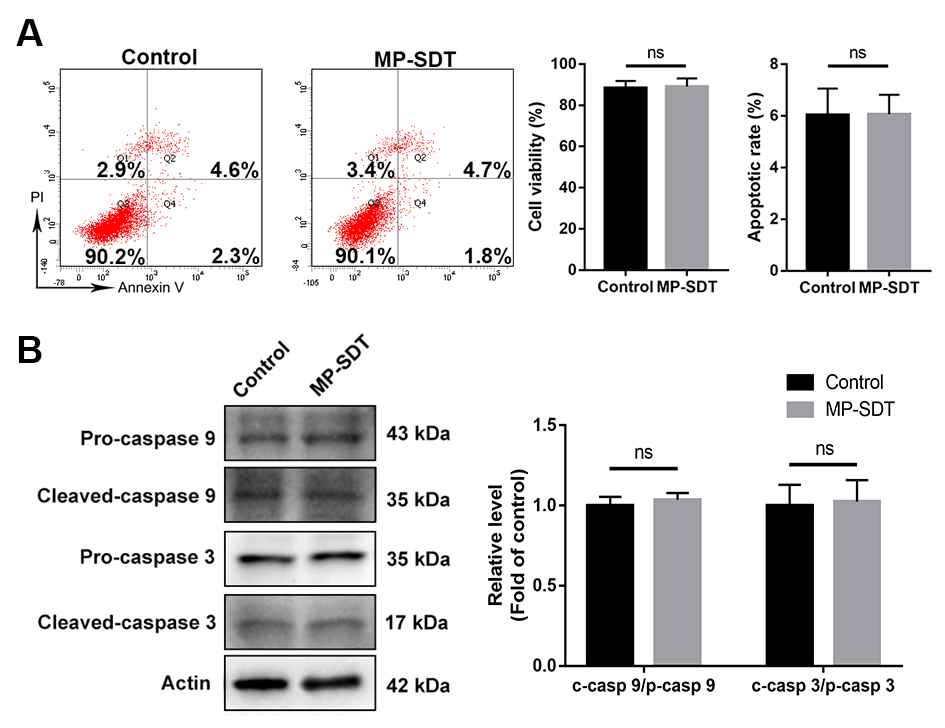

Supplement: S2 Fig — (A) Cell viability detected by Annexin V/PI flow cytometry analysis and quantitative assay. Annexin V-/PI- represents the viable cells. Annexin V+/PI- and Annexin V+/PI+ represent the apoptotic cells. (B) Representative western blots and relative quantitation of cleaved-caspase 9/pro-caspase 9 and cleaved-caspase 3/pro-caspase 3 after MP-SDT. ns: no significance. (TIF) [file pone.0217511.s002.tif]
